# Pharmacokinetics, pharmacogenetics, and toxicity of co-administered efavirenz and isoniazid

**DOI:** 10.4102/sajhivmed.v26i1.1661

**Published:** 2025-03-18

**Authors:** Jessica Taylor, Gary Maartens, Simiso Sokhela, Nomathemba Chandiwana, Godspower Akpomiemie, Francois Venter, Phumla Sinxadi

**Affiliations:** 1 Division of Clinical Pharmacology, Department of Medicine, Faculty of Health Sciences, University of Cape Town, Cape Town, South Africa; 2 Ezintsha, Wits Health Consortium, Faculty of Health Sciences, University of the Witwatersrand, Johannesburg, South Africa; 3 SAMRC/UCT Platform for Pharmacogenomics Research and Translation, South African Medical Research Council, Cape Town, South Africa

**Keywords:** HIV, isoniazid, efavirenz, *CYP2B6*, neurotoxicity

## Abstract

**Background:**

*CYP2B6* slow metabolisers have higher efavirenz concentrations, which are further increased by isoniazid inhibiting efavirenz’s accessory metabolic pathway.

**Objectives:**

We investigated the association between *CYP2B6* genotype and toxicity in people living with HIV (PLWH) on isoniazid and efavirenz.

**Method:**

We enrolled participants from the efavirenz arm of the ADVANCE trial (reference no.: NCT03122262), who received isoniazid and consented to genotyping. We compared efavirenz concentrations on and off isoniazid, stratified by *CYP2B6* genotype. We explored associations between the *CYP2B6* genotype and efavirenz concentrations on isoniazid; and changes over 24 weeks in lipids, alanine aminotransferase (ALT), fasting plasma glucose (FPG), sleep quality, and Modified Mini Screen (MMS) scores.

**Results:**

A total of 168 participants, median age 31 years, 57% female, had classifiable *CYP2B6* genotypes. Efavirenz concentrations on isoniazid were higher (pseudo-median difference 0.49 µg/mL (95% confidence interval [CI] [0.19–0.91]) and associated with increases in total and high-density lipoprotein (HDL)-cholesterol. *CYP2B6* slow metabolisers had higher efavirenz concentrations on isoniazid than extensive metabolisers (*β* = 1.66 [95% CI 0.98–2.34]). *CYP2B6* slow metabolisers had greater increases in total (*β* = 0.44 mmol/L [95% CI 0.01–0.86]) and HDL-cholesterol (*β* = 0.39 mmol/L [95% CI 0.21–0.57]) than extensive metabolisers. There were no associations between efavirenz concentrations or *CYP2B6* genotype, and change in ALT, FPG, low-density lipoprotein (LDL)-cholesterol, triglycerides, sleep quality, or MMS scores.

**Conclusion:**

*CYP2B6* slow metabolisers on isoniazid and efavirenz had greater efavirenz concentrations and increases in total and HDL-cholesterol. We found no association between *CYP2B6* genotype or efavirenz concentrations and sleep or psychiatric symptoms.

**What this study adds:** *CYP2B6* slow metabolisers on efavirenz and isoniazid experienced greater increases in total cholesterol and HDL-cholesterol, likely driven by higher efavirenz concentrations. There was no association between *CYP2B6* genotype or efavirenz concentrations and worsening sleep quality or neuropsychiatric symptoms during 24 weeks of treatment.

## Introduction

Efavirenz, a non-nucleoside reverse transcriptase inhibitor, is predominantly metabolised via the cytochrome P450 enzyme CYP2B6, with minor contributions from CYP2A6. Loss-of-function genetic polymorphisms in *CYP2B6* confer slower metabolism of efavirenz, resulting in increased concentrations and increased risk of efavirenz-related toxicity.^1,2,3^ People with *CYP2B6* slow metaboliser genotypes have a five fold increased risk of discontinuing efavirenz due to neuropsychiatric symptoms.^4^ Increased risk of drug-induced liver injury, hypercholesterolaemia and dysglycaemia in *CYP2B6* slow metabolisers has also been reported.^2,3,5^ The minor metabolic pathway of efavirenz, CYP2A6, is of increased importance in *CYP2B6* slow metabolisers, to prevent accumulation of efavirenz. Isoniazid, which is widely used in the treatment and prevention of tuberculosis, inactivates CYP2A6 via irreversible suicide inhibition.^6^ When co-administered, efavirenz concentrations in *CYP2B6* slow metabolisers are further increased by isoniazid’s inhibition of CYP2A6.^7,8,9^ Polymorphisms in the *NAT2* gene that confer slow *NAT2* acetylator genotypes and increase isoniazid exposure, further increase efavirenz concentrations.^10,11,12^

Initiation of antiretroviral therapy (ART) and isoniazid preventive therapy (IPT) is currently recommended by the WHO for all people living with HIV (PLWH).^13^ However, the clinical significance of the interaction between isoniazid and efavirenz has not been fully characterised. A small descriptive cohort study reported that patients who are both *CYP2B6* slow metabolisers and *NAT2* slow acetylators are at increased risk of severe neurotoxicity when isoniazid and efavirenz are co-administered.^14^ Prospective data on the risk of neurotoxicity, hepatotoxicity, dyslipidaemia, and dysglycaemia associated with this pharmacokinetic and pharmacogenetic interaction are limited. We investigated the association between *CYP2B6* metaboliser genotype and toxicity in PLWH on isoniazid and efavirenz-based ART. We hypothesised that *CYP2B6* slow metabolisers treated with efavirenz and isoniazid will have increased efavirenz concentrations with an increased risk of efavirenz-related neurotoxicity, hepatotoxicity, dyslipidaemia, and dysglycaemia.

## Research methods and design

### Study design and participants

We conducted a sub-study nested in the ADVANCE study (NCT03122262), a 96-week phase 3, open-label, randomised clinical trial conducted in South Africa.^15^ In ADVANCE, ART-naïve participants were randomised to three different ART regimens, and participants who screened negative for tuberculosis symptoms received 12 months of IPT. Our sub-study included adult participants randomised to the efavirenz arm, who consented to genetic testing, and received isoniazid during the first 24 weeks of the study.

### Clinical and biochemical evaluations

Neuropsychiatric symptoms were screened for at baseline, weeks 4, 12, and 24, using the Modified Mini Screen (MMS), and the change in score was calculated. The MMS consists of 22 questions designed to identify symptoms related to psychiatric disorders.^16^ A higher MMS score predicts a greater likelihood of mental illness. Two questions in the MMS related to post-traumatic stress disorder (PTSD) were removed from the calculation of the total score in our study, as PTSD is not considered drug related but rather related to exposure to a traumatic event.^17^ Sleep quality was assessed at baseline, weeks 4, 12, and 24, using a Likert scale, with higher scores indicating better quality, and changes in sleep quality were calculated.^18^ Fasting lipid profiles at baseline and week 24 were used to calculate the change in total cholesterol, low-density lipoprotein (LDL-cholesterol), high-density lipoprotein (HDL-cholesterol), and triglycerides. Alanine aminotransferase (ALT) measured at baseline, week 12, and week 24 was used to calculate the change in ALT. Fasting plasma glucose (FPG) measured at weeks 4, 12, and 24 was used to calculate the change in fasting glucose. Participants who did not initiate, or who discontinued isoniazid in the first 24 weeks of the study, and women who were pregnant during the first 24 weeks of the study, were excluded from these analyses.

### Pharmacokinetic analysis

Efavirenz mid-dosing interval concentrations (10 h to 20 h post dose) were measured at week 24, week 48, week 60, and week 72. Pharmacokinetic samples were centrifuged, with plasma stored at –80 °C until batch analysis. Efavirenz concentrations were determined by means of a validated assay at the pharmacology laboratory of the University of Cape Town.^19^ For each participant, one efavirenz concentration on isoniazid and one efavirenz concentration off isoniazid was selected. Efavirenz concentrations on isoniazid were preferentially determined from week 24 samples, but when missing or below the level of quantification, week 48 samples were used. Efavirenz concentrations off isoniazid were preferentially determined from week 72 samples, but when missing or below the level of quantification, week 60 concentrations were used. We excluded efavirenz concentrations for participants not on isoniazid at week 24 or 48, or not off isoniazid at week 60 or 72, or who were pregnant or within 6 weeks postpartum at the time of sampling.

### Determination and characterisation of genetic polymorphisms

Genomic sampling was performed at week 36 in consenting participants. Whole blood was stored at –80 °C at the Sydney Brenner Institute for Molecular Biosciences, University of the Witwatersrand, for batch DNA extraction at the end of the ADVANCE trial. The salting out method was used to extract DNA from whole blood samples. Genotyping was performed using the MassARRAY® single nucleotide polymorphism (SNP) genotyping system (Agena Bioscience, San Diego, California, United States) at Inqaba Biotechnical Industries, Pretoria, South Africa. Missing SNP data were not imputed.

The following SNPs were genotyped: *CYP2B6* 516G>T (rs3745274); *CYP2B6* 983T>C (rs28399499); *CYP2B6* 15582C>T (rs4803419); *CYP2A6* 47A>C (rs28399433); *NAT2* 191 G>A (rs1801279); *NAT2* 341 T>C (rs1801280) and *NAT2* 590 G>A (rs1799930). *CYP2B6* metaboliser genotype was assigned as: extensive metaboliser (*CYP2B6* 516GG-983TT-15582CC or *CYP2B6* 516GG-983TT-15582CT), intermediate metaboliser (*CYP2B6* 516GG-983TT-15582TT, *CYP2B6* 516GT-983TT-15582CC, *CYP2B6* 516GG-983CT-15582CC, *CYP2B6* 516GT-983TT-15582CT, or *CYP2B6* 516GG-983CT-15582CT), or slow metaboliser (*CYP2B6* 516TT-983TT-15582CC, *CYP2B6* 516GT-983CT-15582CC, or *CYP2B6* 516GG-983CC-15582CC).^20^ *CYP2A6* metaboliser genotype was assigned as: slow metaboliser (*CYP2A6* 47CC), intermediate metaboliser (*CYP2A6* 47AC) and extensive metaboliser (*CYP2A6* 47AA).^21^ *NAT2* acetylator status was assigned as: slow acetylator (homozygous for the variant allele at any locus, or heterozygous at 2 or more loci); intermediate acetylator (heterozygous at a single locus); or rapid acetylator (no variant allele at any locus).^22^

### Statistical analyses

We summarised categorical data using proportions or percentages, and continuous data by mean (standard deviation [s.d.]) or median (interquartile range [IQR]) as appropriate. Genotyped SNPs were tested for Hardy-Weinberg equilibrium using the Chi-square test, or exact tests, as appropriate. We compared absolute change in total cholesterol, HDL-cholesterol, LDL-cholesterol, triglycerides, FPG, ALT, MMS score, and sleep quality over 24 weeks between *CYP2B6* metaboliser genotypes using the Kruskal-Wallis test. Where Kruskal-Wallis test *P*-values were significant, we performed post-hoc Dunn’s pairwise comparisons with Bonferroni correction for multiple testing. We compared paired efavirenz concentrations on and off isoniazid for the cohort overall, and stratified by *CYP2B6* metaboliser genotype, using the Wilcoxon signed-rank test. We investigated the association between *CYP2B6* genotype and log-transformed efavirenz concentrations on isoniazid using multivariable linear regression. Efavirenz concentration data was log-transformed using the natural logarithm to improve model fit and correct for skewness. The covariates age, weight, CD4, HIV viral load, *CYP2A6* genotype, and *NAT2* genotype were included in the model. We used multivariable linear regression to analyse associations between *CYP2B6* metaboliser genotypes, and absolute change in ALT, total cholesterol, HDL-cholesterol, LDL-cholesterol, triglycerides, FPG, MMS score, and sleep quality at various time points during the first 24 weeks of the study. Multivariable linear regression analyses between natural log-transformed efavirenz concentrations on isoniazid and the same variables listed above were performed. A priori selected covariates adjusted for in the multivariable models were age, sex, weight, CD4 count, HIV viral load, *CYP2A6* genotype, *NAT2* genotype, and the baseline value of the dependent variable. Robust standard errors were obtained for all linear regression models. All data manipulation, statistical analyses, and generation of figures was performed using R software (Version 4.2.2, R Foundation for Statistical Computing, Vienna, Austria).^23^

### Ethical considerations

Ethical approval for this sub-study was obtained from the Human Research Ethics Committee of the University of Cape Town (HREC REF: 801/2022). The study was conducted in accordance with the ethical standards of the institutional research committee and the 1964 Declaration of Helsinki and its later amendments. Participants included in the sub-study provided written informed consent for participation in the main study and genetic testing. All data sets were password protected, and all analyses conducted on a password-protected computer. Participants were referred to by study identification number only, and the master list linking study identification number to personal identifying information was not provided to the sub-study investigators conducting the analyses.

## Results

**FIGURE 1:**
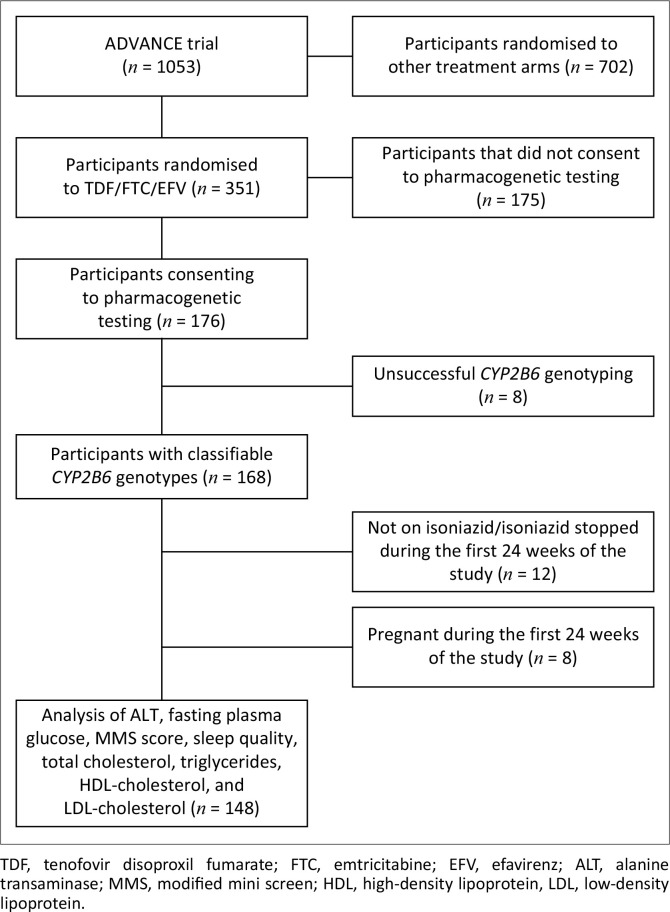
Flow diagram of ADVANCE trial participants enrolled in toxicity and metabolic analyses.

The participant flow is shown in Figure 1 and Online Appendix 1: Figure 1-A1. Of the 176 participants who consented to genetic testing, 168 had classifiable *CYP2B6* genotypes. The baseline characteristics of all participants, categorised by *CYP2B6* metaboliser genotype, are shown in Table 1. Baseline characteristics were similar across *CYP2B6* metaboliser genotypes, except weight, which was lower in *CYP2B6* extensive metabolisers (*P* = 0.02). Of the 148 participants included in the toxicity and metabolic analyses conducted in the first 24 weeks, all were co-prescribed isoniazid for tuberculosis prevention (Figure 1). Of the 77 participants with efavirenz concentrations on isoniazid, 2 participants had been switched from IPT to isoniazid-containing tuberculosis treatment by the time their week 48 efavirenz concentrations were measured (Online Appendix 1: Figure 1-A1).

**TABLE 1:** Baseline characteristics of enrolled participants.

Characteristic	Total				*CYP2B6* metaboliser genotype											
					Extensive				Intermediate				Slow			
	*n*	%	Median	IQR	*n*	%	Median	IQR	*n*	%	Median	IQR	*n*	%	Median	IQR
**Total**	168	100.0	-	-	47	28	-	-	75	45	-	-	46	27	-	-
**Sex**																
Female	96	57.0	-	-	25	53.0	-	-	47	63.0	-	-	24	52.0	-	-
Male	72	43.0	-	-	22	47.0	-	-	28	37.0	-	-	22	48.0	-	-
**Age (years)**	-	-	31	28, 37	-	-	31	26, 37	-	-	31	27, 36	-	-	33	29, 38
**Weight (kg)**	-	-	66	59, 79	-	-	63	58, 69	-	-	68	62, 82	-	-	70	59, 79
**BMI (kg/m**^**2**^)	-	-	23.8	20.3, 27.5	-	-	21.7	19.7, 25.3	-	-	24.3	21.2, 28.3	-	-	24.6	24.6
**CD4 count (cells/μL)**	-	-	295	176, 408	-	-	290	182, 373	-	-	309	187, 420	-	-	291	165, 398
**Log**_**10**_ **viral load (log**_**10**_ **copies/ mL)**	-	-	4.41	3.74, 4.98	-	-	4.42	3.88, 4.80	-	-	4.28	3.51, 5.05	-	-	4.55	3.96, 5.06
***CYP2A6*** **metaboliser genotype**																
Extensive	148	88.0	-	-	45	95.7	-	-	64	85.3	-	-	39	84.8	-	-
Intermediate	17	10.2	-	-	2	4.3	-	-	9	12	-	-	6	13	-	-
Slow	3	1.8	-	-	0	0.0	-	-	2	2.7	-	-	1	2.2	-	-
***NAT2*** **acetylator genotype**																
Rapid	78	46.4	-	-	21	44.7	-	-	37	49.3	-	-	20	43.5	-	-
Intermediate	30	17.9	-	-	10	21.3	-	-	11	14.7	-	-	9	19.5	-	-
Slow	57	33.9	-	-	16	34.0	-	-	24	32.0	-	-	17	37.0	-	-
Unclassifiable	3	1.8	-	-	0	0.0	-	-	3	4.0	-	-	0	0.0	-	-

Note: Height (cm) in mean ± standard deviation: Total: 169 ± 9 cm; *CYP2B6* metaboliser genotype (extensive: 169 ± 8; Intermediate 169 ± 9; Slow: 169 ± 10).

IQR, interquartile range; BMI, body mass index; *n*, number of participants.

### Genotyping

All genotyped SNPs were in Hardy-Weinberg equilibrium, except *CYP2A6* 47 A>C (rs28399433) (*P* = 0.03). Minor allele frequencies are reported in the Online Appendix 1: Table 1-A1. The *CYP2B6* metaboliser genotype frequencies were: 28% extensive, 45% intermediate, and 27% slow. The *CYP2A6* metaboliser genotype frequencies were: 88% extensive, 10% intermediate, and 2% slow. The *NAT2* acetylator genotype frequencies were: 46% rapid, 18% intermediate, 34% slow, and 2% unclassifiable. In three participants, *NAT2* acetylator status could not be classified because of genotyping failure.

### Pharmacokinetic analysis

**FIGURE 2:**
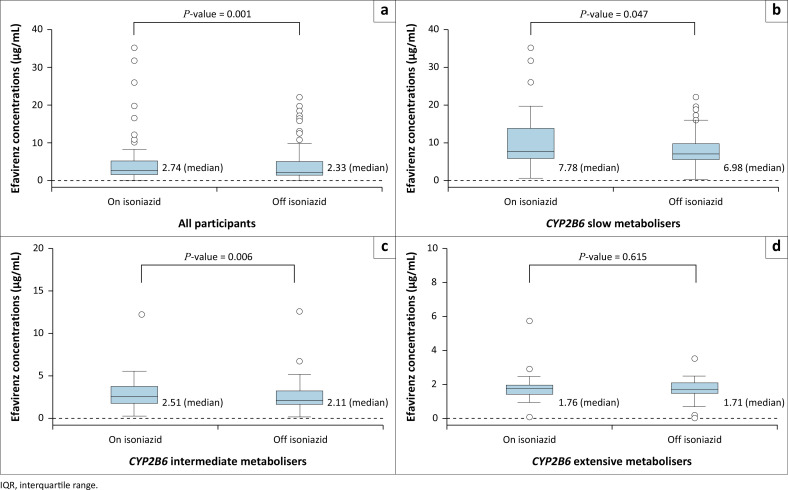
Median (IQR) efavirenz concentrations on and off isoniazid in: (a) All participants, (b) *CYP2B6* slow metabolisers, (c) *CYP2B6* intermediate metabolisers, and (d) *CYP2B6* extensive metabolisers. Concentrations compared using the Wilcoxon signed-rank test for paired samples.

**FIGURE 3:**
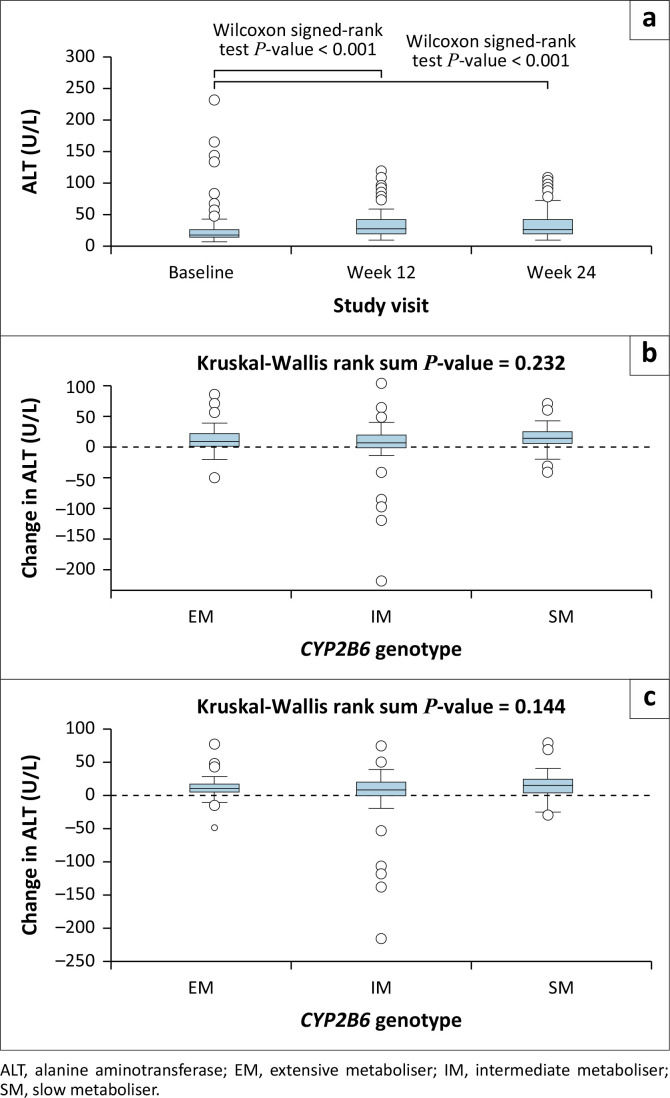
(a) Alanine aminotransferase (ALT) at baseline, week 12 and week 24 of the study for cohort; (b) change in ALT from baseline to week 12 by genotype; (c) change in ALT from baseline to week 24 by genotype.

In 77 participants on isoniazid, the median plasma mid-dose efavirenz concentration was 2.74 µg/mL (IQR 1.76–5.40 µg/mL). Off isoniazid, in 146 participants, the median plasma mid-dose efavirenz concentration was 2.33 µg/mL (IQR 1.67–5.12 µg/mL). Efavirenz concentrations were greater while on isoniazid, compared to off isoniazid, for the cohort overall (Wilcoxon signed-rank test for paired samples: pseudo-median difference 0.49 µg/mL (95% CI [confidence interval] 0.19–0.91) (Figure 2a). When stratified by genotype, this difference was largest in *CYP2B6* slow metabolisers (Wilcoxon signed-rank test for paired samples: pseudo-median difference 2.13 µg/mL [95% CI 0.02–8.77]) (Figure 2b). A significant difference was also found in *CYP2B6* intermediate metabolisers (Wilcoxon signed-rank test for paired samples: pseudo-median difference 0.52 µg/mL [95% CI 0.16–0.89]), but not *CYP2B6* extensive metabolisers (Wilcoxon signed-rank test for paired samples: pseudo-median difference 0.07 µg/mL [95% CI –0.25–0.39]) (Figure 2c and d). In multivariable linear regression, only *CYP2B6* slow metabolisers and body weight were associated with efavirenz concentrations on isoniazid (Online Appendix 1: Table 2-A1). *CYP2B6* slow metabolisers on isoniazid had higher efavirenz concentrations than extensive metabolisers (*β* = 1.66 [95% CI 0.98–2.34], *P* < 0.001) (Online Appendix 1: Table 2-A1). We found no association between *NAT2* acetylator or *CYP2A6* metaboliser genotype and efavirenz concentrations, both while on isoniazid in univariable analyses, and after adjusting for *CYP2B6* genotype. Of the 46 *CYP2B6* slow metabolisers in our cohort, one was a *CYP2A6* slow metaboliser and six were *CYP2A6* intermediate metabolisers. The participant who was both a *CYP2B6* and *CYP2A6* slow metaboliser did not have a measured efavirenz concentration while on isoniazid. Three of six *CYP2B6* slow and *CYP2A6* intermediate metabolisers had measured efavirenz concentrations on isoniazid. These concentrations ranged from 26–35.20 µg/mL. In contrast, efavirenz concentrations in *CYP2B6* slow and *CYP2A6* extensive metabolisers ranged from 0.69–19.8 µg/mL (Online Appendix 1: Figure 2-A1). In *CYP2B6* slow metabolisers, there was no difference in efavirenz concentrations on isoniazid by *NAT2* acetylator genotype (Online Appendix 1: Figure 3-A1).

### Alanine aminotransferase

There were 103 adverse events of elevated ALT reported in 68 participants from our cohort during 96 weeks of the ADVANCE trial (Online Appendix 1: Table 3-A1). According to the division of AIDS (DAIDS) table for grading the severity of adult adverse events: 69 events were of grade 1 severity, 24 events of grade 2 severity, 9 events of grade 3 severity, and 1 event was of grade 4 severity.^24^ Significantly more elevated ALT adverse events occurred in *CYP2B6* intermediate (38.8%) and slow metabolisers (38.8%), than in extensive metabolisers (22.3%, *P* = 0.02). There was an association between *CYP2B6* metaboliser genotype and severity of ALT elevation (*P* = 0.03) (Online Appendix 1: Table 3-A1). Grade 2 ALT elevations occurred more frequently in *CYP2B6* slow metabolisers than in intermediate or extensive metabolisers (*P* = 0.003 and *P* < 0.001, respectively). ALT increased from baseline to weeks 12 and 24 (Figure 3a), but there was no difference in the magnitude of increase by *CYP2B6* metaboliser genotype (Figures 3b and c). In multivariable linear regression, there was no association between *CYP2B6* slow metabolisers and change in ALT from baseline to week 12 or 24 (Online Appendix 1: Table 4-A1 and Table 5-A1). There was no association between efavirenz concentrations on isoniazid and change in ALT from baseline to week 12 or 24 (Online Appendix 1: Table 6-A1).

### Fasting plasma glucose

In our cohort, one participant developed diabetes, and four developed impaired glucose tolerance during 96 weeks of the ADVANCE trial. FPG increased from baseline to weeks 4, 12, and 24. There was no difference in the magnitude of increase in FPG from baseline to these study time points between *CYP2B6* slow, intermediate, and extensive metabolisers. In multivariable linear regression, the *CYP2A6* slow metaboliser genotype was associated with a decrease in FPG from baseline to week 12 (*β* = –0.72 mmol/L [95% CI –1.40 to –0.03], *P* = 0.04) (Online Appendix 1: Table 7-A1). We found no association between efavirenz concentrations on isoniazid, and change in FPG from baseline to weeks 4, 12, and 24 (Online Appendix 1: Table 8-A1).

### Lipids

**FIGURE 4:**
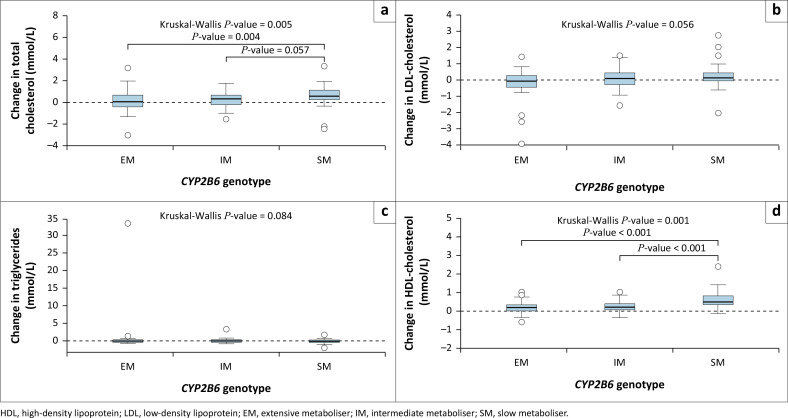
Change in (a) total cholesterol, (b) LDL-cholesterol, (c) triglycerides, and (d) HDL-cholesterol from baseline to week 24 by *CYP2B6* genotype. Where Kruskal-Wallis test *P*-values were significant, post-hoc Dunn’s pairwise comparisons with Bonferroni correction for multiple testing were performed.

Thirty-six adverse events relating to hypercholesterolemia, 17 adverse events relating to elevated LDL-cholesterol, and 19 adverse events relating to hypertriglyceridaemia, were reported in our cohort for the 96-week duration of the ADVANCE trial. There was no association between *CYP2B6* metaboliser genotype and the number or severity of these adverse events (Online Appendix 1: Table 9-A1, Table 10-A1, Table 11-A1). Total cholesterol and HDL-cholesterol increased from baseline to week 24. The magnitude of these increases was greatest in *CYP2B6* slow metabolisers (Figure 4a and Figure 4d). *CYP2B6* slow metabolisers experienced 0.44 mmol/L greater increase in total cholesterol, and 0.39 mmol/L greater increase in HDL-cholesterol, as compared to extensive metabolisers (Table 2). Similarly, each log increase in efavirenz concentration on isoniazid was associated with a 0.18 mmol/L increase in both total (*β* = 0.18 mmol/L [95% CI 0.03–0.33]) and HDL-cholesterol (*β* = 0.18 mmol/L [95% CI 0.06–0.30]) (Online Appendix 1: Table 12-A1). There was no increase in LDL-cholesterol from baseline to week 24 (Wilcoxon signed-rank test for paired samples: pseudo-median difference 0.08 mmol/L [95% CI –0.01–0.16], *P* = 0.08) for the cohort overall. However, when stratified by genotype, in *CYP2B6* slow metabolisers there was a trend toward a significant increase in LDL-cholesterol over 24 weeks (Figure 4b). In multivariable regression, the *CYP2B6* slow metaboliser genotype was associated with a greater increase in LDL-cholesterol, but this was not statistically significant (*β* = 0.27 mmol/L [95% CI –0.05–0.59], *P* = 0.09) (Table 2). We found no association between efavirenz concentrations on isoniazid and change in LDL-cholesterol over 24 weeks (*β* = 0.002 mmol/L [95% CI –0.11–0.12]) (Online Appendix 1: Table 12-A1). Triglycerides decreased from baseline to week 24 (Wilcoxon signed-rank test for paired samples: pseudo-median difference –0.09 mmol/L [95% CI –0.16 to –0.02], *P* = 0.01). There was no difference in the change in triglycerides over 24 weeks by metaboliser genotype (Figure 4c). There was no association between *CYP2B6* slow metabolisers and change in triglycerides in multivariable linear regression (Table 2). There was also no association between efavirenz concentrations on isoniazid and change in triglycerides (Online Appendix 1: Table 12-A1).

**TABLE 2:** Multivariable linear regression models for change in total cholesterol, LDL-cholesterol, HDL-cholesterol and triglycerides from baseline to week 24.

Covariates	Change in total cholesterol (mmol/L) (baseline to week 24)†			Change in LDL-cholesterol (mmol/L) (baseline to week 24)‡			Change in HDL-cholesterol (mmol/L) (baseline to week 24)§			Change in triglycerides (mmol/L) (baseline to week 24)¶		
	*β*	95% CI	*P*	*β*	95% CI	*P*	*β*	95% CI	*P*	*β*	95% CI	*P*
***CYP2B6*** **genotype**												
Extensive metaboliser	Ref	Ref	Ref	Ref	Ref	Ref	Ref	Ref	Ref	Ref	Ref	Ref
Intermediate metaboliser	0.13	−0.21 to 0.47	0.45	0.19	−0.08 to 0.45	0.15	0.06	−0.06 to 0.18	0.36	−0.76	−2.59 to 1.07	0.41
Slow metaboliser	0.44	0.01 to 0.86	0.04¢	0.27	−0.05 to 0.59	0.09	0.39	0.21 to 0.57	<0.001¢	−1.50	−4.59 to 1.60	0.34
***CYP2A6*** **genotype**												
Extensive metaboliser	Ref	Ref	Ref	Ref	Ref	Ref	Ref	Ref	Ref	Ref	Ref	Ref
Intermediate metaboliser	0.04	−0.30 to 0.38	0.82	−0.11	−0.39 to 0.17	0.44	0.17	−0.01 to 0.35	0.06	−0.23	−0.95 to 0.49	0.53
Slow metaboliser	0.23	−0.39 to 0.85	0.47	0.09	−0.90 to 1.08	0.85	−0.08	−0.42 to 0.26	0.64	0.07	−2.40 to 2.54	0.96
***NAT2*** **genotype**												
Rapid acetylator	Ref	Ref	Ref	Ref	Ref	Ref	Ref	Ref	Ref	Ref	Ref	Ref
Intermediate acetylator	0.21	−0.21 to 0.62	0.33	0.21	−0.17 to 0.60	0.27	0.02	−0.20 to 0.25	0.85	0.72	−1.32 to 2.77	0.49
Slow acetylator	0.25	−0.22 to 0.72	0.29	0.07	−0.31 to 0.45	0.70	0.13	−0.10 to 0.37	0.26	1.16	−1.85 to 4.16	0.45
**Sex**												
Female	Ref	Ref	Ref	Ref	Ref	Ref	Ref	Ref	Ref	Ref	Ref	Ref
Male	−0.26	−0.56 to 0.03	0.08	−0.27	−0.51 to -0.03	0.03¢	−0.14	−0.27 to -0.01	0.03¢	0.08	−0.38 to 0.55	0.71
Age (years)	0.01	−0.01 to 0.04	0.23	0.01	−0.01 to 0.03	0.19	0.002	−0.01 to 0.01	0.73	0.05	−0.06 to 0.17	0.37
Baseline CD4 (per 100 cells)	−0.02	−0.12 to 0.09	0.73	−0.06	−0.13 to 0.02	0.14	−0.02	−0.05 to 0.01	0.22	0.26	−0.30 to 0.83	0.36
Baseline VL (log_10_ cp/mL)	−0.02	−0.21 to 0.18	0.87	−0.04	−0.21 to 0.12	0.63	−0.03	−0.10 to 0.04	0.41	0.27	−0.23 to 0.77	0.29
Weight (kg)	−0.003	−0.01 to 0.01	0.51	0.002	−0.01 to 0.01	0.69	−0.003	−0.01 to 0.002	0.26	−0.02	−0.06 to 0.03	0.46
Baseline value of dependent variable (mmol/L)	−0.45	−0.67 to -0.22	<0.001¢	−0.50	−0.73 to -0.26	< 0.001¢	−0.17	−0.29 to -0.04	0.01¢	1.45	−2.97 to 5.86	0.52

LDL, low-density lipoprotein; HDL, high-density lipoprotein; 95%CI, 95% confidence interval; *NAT2*, N-acetyltransferase 2; VL, viral load; cp, copies; kg, kilograms; *df*, degrees of freedom.

¢ , statistically significant i.e. *p* < 0.05.

† , Model adjusted R-squared: 0.194; *F*-statistic 2.338 on 12 and 129 d*f*; P = 0.01; 26 observations deleted due to missing data;

‡ , Model adjusted R-squared: 0.2935; F-statistic 1.818 on 12 and 129 *df; P* = 0.05; 26 observations deleted due to missing data;

§ , Model adjusted R-squared: 0.2472; *F*-statistic 4.589 on 12 and 129 *df; P* < 0.001; 26 observations deleted due to missing data;

¶ , Model adjusted R-squared: 0.05214; *F*-statistic 0.161 on 12 and 129 *df; P* = 0.9994; 26 observations deleted due to missing data.

### Modified mini screen

Twenty-five psychiatric adverse events were reported in 22 participants from our cohort during the 96-week ADVANCE trial. All were of grade 1 (mild) or grade 2 (moderate) severity. There was no association between *CYP2B6* metaboliser genotype and the number of psychiatric adverse events. At baseline, the median MMS score of participants was 0. MMS score improved over 24 weeks (Wilcoxon signed-rank test for paired samples: pseudo-median difference –1.00 [95% CI –1.50 to –0.999] *P* < 0.001). There was no association between *CYP2B6* genotype or efavirenz concentrations on isoniazid and change in MMS score from baseline to week 4, 12, or 24 in multivariable linear regression models (Online Appendix 1: Table 13-A1 and Table 14-A1).

### Sleep score

At baseline, the median sleep quality score was 8. There was an improvement in sleep quality over 24 weeks to a median score of 9 (Wilcoxon signed-rank test for paired samples: pseudo-median difference 1.50 [95% CI 1.00–1.5], *P* < 0.001). When stratified, there was no significant difference by *CYP2B6* genotype, in the improvement in sleep quality from baseline to week 4, 12, or 24. There was no association between *CYP2B6* slow metabolisers and change in sleep score from baseline to week 4, 12, or 24 in multivariable linear regression models (Online Appendix 1: Table 15-A1). There was also no association between efavirenz concentrations on isoniazid and change in sleep score from baseline to week 4,12, or 24 (Online Appendix 1: Table 16-A1).

## Discussion

In this sub-study of a randomised controlled trial, we found that *CYP2B6* slow metabolisers had higher efavirenz concentrations and increased total and HDL-cholesterol during the first 24 weeks of treatment with efavirenz and isoniazid. We found no association between *CYP2B6* slow metabolisers and increases in ALT, FPG, LDL-cholesterol, or triglycerides. We also found no association between *CYP2B6* slow metabolisers or efavirenz concentrations when on isoniazid, and worsening sleep quality or neuropsychiatric symptoms.

We found that efavirenz concentrations were increased when co-administered with isoniazid. The increase in efavirenz concentrations is most marked in *CYP2B6* slow metabolisers, but also significant in *CYP2B6* intermediate metabolisers, consistent with previous studies.^7,9,11,25^ We found no association between *NAT2* slow acetylators and increased efavirenz concentrations while on isoniazid in all participants, nor in *CYP2B6* slow metabolisers specifically, contrasting with other studies.^10,11,12,25,26,27^ This may be because our ability to classify *NAT2* acetylator status accurately was limited by the inclusion of only three SNPs, rather than all SNPs associated with slow acetylation.^22,28^ Our study also had limited power to detect smaller effects of *NAT2* slow acetylators on efavirenz concentrations.

We found no association between *CYP2A6* slow metabolisers and increased efavirenz concentrations overall, and after adjusting for *CYP2B6* genotype. However, *CYP2B6* slow metabolisers with concomitant *CYP2A6* intermediate metaboliser genotypes had greater efavirenz concentrations on isoniazid, than *CYP2B6* slow metabolisers with *CYP2A6* extensive metaboliser genotypes. Earlier studies that failed to show any association between *CYP2A6* 47A>C (rs28399433) and increased efavirenz concentrations in *CYP2B6* slow metabolisers were limited by too few participants homozygous for the variant allele, or too few *CYP2B6* slow metabolisers.^29,30^ Our results highlight the crucial role of the accessory metabolic pathway, CYP2A6, in preventing efavirenz accumulation in *CYP2B6* slow metabolisers previously suggested.^27^

The association between *CYP2B6* slow metabolisers and increases in total cholesterol and HDL-cholesterol we report is consistent with the known adverse effect profile of efavirenz.^31,32^ It has been suggested that the increase in HDL-cholesterol after starting efavirenz-based ART is a ‘return to health’ phenomenon, since low HDL-cholesterol is the most common lipid abnormality associated with untreated HIV.^33^ In our cohort overall, HDL-cholesterol increased over 24 weeks. This increase was most marked in *CYP2B6* slow metabolisers, and our analyses of the association between efavirenz concentrations and total and HDL-cholesterol confirm a concentration-dependent effect of efavirenz.^34^ Efavirenz-mediated induction of pregnane X receptor (PXR) transcriptional activity stimulates fatty acid uptake and cholesterol biosynthesis in hepatocytes, while simultaneously suppressing the expression of a major receptor for HDL-cholesterol uptake in the liver, leading to increases in total cholesterol, LDL-cholesterol, and HDL-cholesterol.^34^ These effects are not unique to efavirenz, as activation of PXR has been shown to mediate hypercholesterolaemia associated with various other drugs.^35^ Associations between efavirenz concentrations and increasing total cholesterol, LDL-cholesterol, HDL-cholesterol, and triglycerides have been reported in a South African cohort.^2^ Our current study supports these previous findings with respect to total and HDL-cholesterol. Increases in HDL-cholesterol are considered protective against cardiovascular disease and mortality, and are therefore not necessarily a drug-related ‘toxicity’.^36^ However, pharmacological treatments aimed at increasing HDL-cholesterol specifically have not been associated with reduction in mortality, and observational data provide evidence that very high HDL-cholesterol concentrations may paradoxically increase mortality.^36,37,38^ These increases in HDL-cholesterol can therefore not be considered completely benign, especially in those with normal or elevated HDL-cholesterol at baseline.

There was no association between *CYP2B6* slow metabolisers and increased FPG in our study, which contrasts with a case-control study reporting a four-fold increase in the risk of diabetes associated with the *CYP2B6* 516G>T SNP.^5^ Our previous work also found no association between *CYP2B6* metaboliser genotypes and FPG, despite reporting an association between efavirenz concentrations and FPG which was also not reproduced in this current study.^2^ The association between *CYP2A6* slow metabolisers and a decrease in FPG over 12 weeks in our present study is unexpected and unexplained, and may be due to chance.

We found no association between *CYP2B6* slow metabolisers or efavirenz concentrations and ALT in PLWH on isoniazid and efavirenz, suggesting that efavirenz-related hepatotoxicity is not concentration-dependent. Previous studies have reported associations between increasing efavirenz concentrations or *CYP2B6* slow metabolisers, and hepatic adverse events or liver enzyme elevations when considered as a binary outcome.^3,39^ Although we found no significant association between *CYP2B6* slow metabolisers and change in ALT over 12 and 24 weeks, we did find that the largest proportion of ALT-related adverse events in our cohort occurred in slow and intermediate *CYP2B6* metabolisers. Most of these events were mild and occurred during the first 48 weeks of the study when participants would have been co-prescribed isoniazid and efavirenz. Retrospective data suggest that efavirenz drug-induced liver injury presents after a median duration of 6 months of treatment.^40,41^ By only investigating changes in ALT from baseline to week 12 and week 24, our study may have missed increases in ALT occurring outside these time points.

Despite the known pharmacokinetic interaction of efavirenz and isoniazid, we found no association between *CYP2B6* metaboliser genotype or efavirenz concentrations and neuropsychiatric symptoms in PLWH co-prescribed both drugs. An AIDS Clinical Trials Group study reported an association between *CYP2B6* 516G>T SNP and neuropsychiatric symptoms within 1 week of efavirenz treatment, but not later, suggesting the development of tolerance to these adverse effects. Similarly, a South African study reported resolution of the early neuropsychiatric adverse effects of efavirenz within 1 month of treatment – by only screening for symptoms from week 4 onward, we may have missed any early associations.^42^ Alternatively, neuropsychiatric effects of the interaction between efavirenz and isoniazid may be the result of chronic exposure to or the accumulation of toxic metabolites. In a study of 15 participants with late-onset efavirenz neurotoxicity (LENS), all were *CYP2B6* slow metabolisers, and either *NAT2* slow or intermediate acetylators.^14^ The median duration of efavirenz-based ART prior to admission for LENS was 2.2 years. Most of this cohort also received isoniazid (87%), with the median duration of isoniazid therapy prior to presentation of 13 months.^14^ In another South African cohort of patients with LENS, the median duration of ART prior to presentation was 2.8 years, and for IPT was 6 months.^43^ As we only studied changes in neuropsychiatric symptom scores up to week 24, we cannot exclude an association beyond this period.

A key strength of our study is the ability to examine the pharmacokinetic, pharmacogenetic, and pharmacodynamic components of the efavirenz-isoniazid interaction within the same cohort, utilising data collected prospectively as part of a randomised controlled trial. However, our study has some limitations. Our small sample size may have limited power to detect significant associations between efavirenz concentrations or *CYP2B6* slow metabolisers and smaller changes in metabolic, hepatic, and neuropsychiatric effects. Our analyses of the association between efavirenz concentrations on isoniazid, and change in ALT, FPG, lipids, MMS score, and sleep score were further limited by missing concentration data. Finally, since we only compared changes in variables during the first 24 weeks, while all metaboliser genotypes were co-prescribed efavirenz and isoniazid, we were unable to fully characterise the impact of the addition of isoniazid on toxicity alone.

## Conclusion

In conclusion, among South African PLWH on efavirenz-based ART and isoniazid, *CYP2B6* slow metabolisers experienced greater increases in total cholesterol and HDL-cholesterol as compared to extensive metabolisers, driven by higher plasma efavirenz concentrations. Despite these elevated efavirenz concentrations while on treatment with isoniazid and efavirenz, we found no association between *CYP2B6* slow metabolisers or efavirenz concentrations on isoniazid and worsening sleep quality or neuropsychiatric symptoms. We found no association between *CYP2B6* genotype or efavirenz concentrations on isoniazid, and dysglycaemia, or elevations in ALT, triglycerides or LDL-cholesterol.
